# Trauma, Sleep and Mental Health Problems in Low-Income Young Adults

**DOI:** 10.3390/ijerph18031145

**Published:** 2021-01-28

**Authors:** Rachel A. Fusco, Yan Yuan, Hyunji Lee, Christina E. Newhill

**Affiliations:** 1 School of Social Work, University of Georgia, Athens, GA 30602, USA; 2 School of Social Work, University of Pittsburgh, Pittsburgh, PA 15260, USA; YAY57@pitt.edu (Y.Y.); Hyunji.lee@pitt.edu (H.L.); Newhill@pitt.edu (C.E.N.)

**Keywords:** anxiety, depression, trauma exposure

## Abstract

Low-income young adults are more likely to have exposure to trauma, which increases risk for mental health problems. Although adequate sleep promotes good health, people with histories of trauma are more likely to have sleep problems. The current study explored whether poor sleep mediated the relationship between trauma exposure and mental health. A sample of 143 low-income 18–24-year-old young adults completed depression, anxiety, and trauma exposure measures and wore sleep monitors for four nights. Structural equation modeling (SEM) was used to examine both direct and indirect effects of variables. Results showed that higher trauma exposure was associated with depression and anxiety. Mean sleep hours per night was fewer than six, far below recommended guidelines for optimal health and functioning. Fewer sleep hours partially mediated the relationship between both trauma exposure and depression and anxiety, and the direct effect from trauma remained significant after adjusting for the partial mediation from sleep.

## 1. Introduction

A growing body of evidence shows that sleep plays a critical role in overall health. Poor sleep has been associated with a range of problems such as obesity, violence and aggression, and impaired cognitive functioning [1,2,3]. People in young adulthood (defined as those aged 18–25 years) may be especially likely to get insufficient sleep and suffer from subsequent negative outcomes [4]. There are both biological and behavioral reasons that young adults are more sleep deprived. Young adults experience hormonal changes that can disrupt sleep, and a decrease in synaptic density affecting the brain’s structure and organization can impact sleep [5].

Subpopulations of young adults such as those in poverty may be at greater risk for sleep problems [6]. They may also be more at risk for trauma exposure and subsequent mental health problems [7]. This suggests a complex relationship between trauma exposure, mental health, life challenges, and sleep problems in young adults.

### 1.1. Trauma and Mental Health

Trauma exposure has been consistently linked with higher risk for developing mental health difficulties. For example, a large body of literature reports a significant relationship between early trauma experiences and problems such as attachment disruptions and social impairments, dissociation, and emotion dysregulation [8,9,10]. Studies that are more recent have focused on understanding the influence of multiple trauma types on mental health outcomes as opposed to focusing on one specific form of trauma [11].

Studies have also investigated factors that further amplify the association between traumatic exposure and mental health difficulties, such as socioeconomic status and race/ethnicity. For example, a study reported that Hispanic and non-Hispanic Black youth experience higher rates of multiple victimization relative to their White counterparts [12]. Experiencing multiple victimizations not only significantly predicted mental health disorders after accounting for individual trauma types but also showed a stronger effect in terms of predicting disorders compared to multiple exposures to a single type of trauma [13]. Given the higher rates of multiple victimization reported among racial/ethnic minority individuals, subsequent problems facing them may be more severe and complex.

In addition to racial/ethnic disparities, members of lower socioeconomic status groups suffer from higher rates of trauma and related mental health consequences. In particular, people with low incomes, low education levels, and/or low occupational status more frequently experience traumatic events, which are related to a wide array of mental health disorders [7]. Having a low income and limited access to education show the strongest effects on trauma disparities among younger populations [14].

### 1.2. Sleep

The ability to initiate and maintain sleep is closely related to aspects of stress regulation and vigilance [15]. One element of trauma theory as conceptualized by Herman [16] is that exposure to traumatic events can leave the brain in a perpetually hyperaroused state. Individuals who experience high levels of vigilance or who perceive their environment as unsafe are therefore vulnerable to sleep disturbances [17]. Sleep disruptions have been strongly associated with psychiatric problems, especially depression and anxiety, in community samples of young adults [18].

Sleep disturbances are frequently associated with mental health problems, most commonly depression [19]. Insomnia lasting more than two weeks was predictive of major depressive disorder in a longitudinal study of young adults [20]. Because depressive and anxiety disorders are highly co-morbid, it is difficult to understand the unique relationship that each might have with sleep problems [21]. While the relationship between sleep and mental health is well established, there are still many unanswered questions about the etiology.

Previous research has demonstrated significant associations among poor sleep, trauma, and mental health problems in young adults [22,23]. A systematic review of the effects of adverse childhood experiences (ACEs) on sleep disorders among adults concluded that a higher number of ACEs was associated with a longer sleep onset latency and a shorter period of staying asleep [23]. Specifically, early adverse experiences and post-traumatic stress disorder have both been associated with poor health habits and health outcomes, including smoking and sleep disturbances [22].

The negative effects of childhood trauma on sleep problems have been evidenced in clinical and subclinical populations. For example, Hamilton and colleagues [24] found that childhood maltreatment strongly predicted insomnia, controlling for current depression, anxiety, and low socioeconomic status. Another study of 161 adults reporting childhood trauma found that the effects of early life traumatic experiences on sleep quantity and quality likely persist into adulthood [25]. Trauma exposure in adulthood, however, did not predict sleep problems, underscoring the strong association between early life traumas and sleep health.

Some studies have tested whether sleep problems predict mental health outcomes in young adults [26]. A study of young adults aged from 17 to 24 years in Australia showed that the significant association between shorter sleep duration and higher levels of self-reported psychological distress remained at a 1-year follow-up after controlling for a range of other potential explanatory factors [27]. A recent study of preadolescent children showed that sleep problems mediated the effects of ACEs on externalizing problems after controlling for demographics, post-traumatic stress, and use of psychotropic medication [28]. These studies highlight the role of sleep problems in mediating associations between trauma exposure and mental health among young adults at high risk.

The primary aim of the current study is to better understand the role of sleep in the lives of low-income young adults, particularly the relationship of sleep with both trauma exposure and mental health, by examining three research questions: (1) What is the level of trauma exposure in low-income young adults? (2) What are the sleep patterns of low-income young adults? (3) Does sleep mediate the relationship between trauma exposure and mental health outcomes?

## 2. Materials and Methods

### 2.1. Participants

Young adults between 18 and 24 years old (*N* = 143) were recruited from a local agency in a U.S. city that provides support and workforce development training for low-income young adults under age 25. Any young adult between 18 and 24 earning below the U.S. poverty guideline of USD 12,760 per year was eligible for services. The agency offers both structured groups and drop-in meetings with mentors, and young adults usually visit the agency at least once a week for a 3–6-month period. Participants recruited for the study needed to have a regular place to sleep to be eligible for study enrollment.

### 2.2. Procedures

Fliers providing study information were posted in common areas of the agency where young adults meet with friends, watch TV, and access free laundry services. Interested young adults could phone, email, or text the study team for more information. Individuals with diagnosed sleep disorders or who reported taking medication (either prescription or over-the-counter) for sleep were excluded from the study. Participants received USD 100 compensation for survey completion and wearing a Fitbit monitor for five consecutive nights. Informed consent was obtained from all study participants and the University of Pittsburgh Institutional Review Board approved the research procedures.

### 2.3. Measures

#### 2.3.1. Sleep

Sleep was measured with a Fitbit Charge watch, which participants wore for five days. The Fitbit Charge watches collected data on total time asleep, length of time to fall asleep (sleep onset latency), and number of awakenings. These factors are all considered important quantitative measures of sleep problems. Fitbit Charge has shown effectiveness in measuring sleep time in prior studies [29]. A study comparing the Fitbit Charge to polysomnography found that the Fitbit watch slightly overestimated total sleep time and sleep efficiency and underestimated wake after sleep onset by 5 min in a sample of adolescents [30]. Fitbits have high inter-device reliability [31]. Data were downloaded and analyzed with SPSS, and there were no missing data.

#### 2.3.2. Depression

The Patient Health Questionnaire (PHQ) is a nine-item scale that asks respondents to rate depressive symptoms over the past two weeks. Responses to items range from 0 (Not at all) to 3 (Nearly every day) for a total score from 0 to 27 [32]. In clinical settings, major depression is diagnosed if five or more symptom criteria have been present for at least “more than half the days” in the past two weeks, and one of the symptoms is depressed mood or anhedonia. The PHQ-9 has been found to have construct validity not only in medical settings but in the general population [33,34].

#### 2.3.3. Anxiety

The Beck Anxiety Inventory (BAI) is a self-report measure with 21 multiple-choice items in which the respondent is asked to rate the severity of various anxiety symptoms across the past week [35]. Each symptom is presented with four responses (not at all, mildly, moderately, and severely) and the respondent is asked to endorse the one that best applies. A score above 10 indicates mild anxiety, and a score above 19 indicates moderate anxiety [36]. The BAI has high internal consistency, test–retest reliability, and good concurrent and discriminant validity. It is widely used in research and to screen for anxiety disorders in a range of populations [37,38].

#### 2.3.4. Trauma Exposure

Trauma exposure was measured with the Brief Trauma Questionnaire (BTQ), a screening tool to determine whether an individual has experienced a traumatic event that meets the criteria required for a diagnosis of Posttraumatic Stress Disorder (PTSD) [39]. The measure evaluates 10 potentially traumatic life events including severe physical abuse, serious motor vehicle accidents, death of a close friend or family member, and life-threatening illnesses. Kappa coefficients for all event-specific items range from 0.74 to 1.00 [40]. Interrater reliability has been shown to be good to excellent for all of the trauma categories, and criterion validity has been demonstrated repeatedly, with expected associations found between BTQ measured trauma and PTSD symptomology [41]. Scores range from 0 to 10.

### 2.4. Data Analysis

Descriptive statistical analyses were conducted to show participant characteristics across variables. Means and standard deviations were calculated for the depression, anxiety, and trauma exposure scales. Mean sleep hours, sleep onset latency, and number of nighttime awakenings were also calculated and were used as indicators in the measurement model of our structural equation modeling (SEM).

Additional descriptive statistics such as simple correlations among the variables of interest, *t*-tests, and a multivariate analysis of variance (MANOVA) were fully examined before advanced statistical analyses were conducted. After initial examination of the simple correlations, a multiple regression analysis without interaction terms was conducted in R, where sleep hours was the outcome variable and mental health outcomes were the primary predictors.

Finally, a multi-level mediation model was created using the structural equation modeling method (SEM) in R, using trauma history as the predictor, sleep as the mediator, and depression scores and anxiety scores as the outcome variables. SEM is an analytical approach that allows for testing theoretical models comprising both direct and indirect effects [42]. A direct effect concerns a regression-type relationship between the independent variable and dependent variable (e.g., Trauma → Depression/Anxiety), whereas an indirect one deals with a relationship between those two variables mediated/intervened through a third variable (or a set of other variables) (e.g., Trauma → Sleep → Depression/Anxiety).

Using SEM over traditional regression analyses allows us to model an unobserved, abstract concept (i.e., a latent factor of “Sleep”) and to test multiple lines of hypothesized relationships simultaneously while accounting for measurement errors [43]. In light of that, we were able to investigate a dynamic interplay between factors at different levels (from micro- to macroenvironments). To date, there is no consensus in terms of deciding sample sizes of SEM. A past review of SEM studies reported that the median sample size for testing an indirect effect was about 142.5, and about 40% of all the studies included fewer than 150 participants [44]. A common guide that researchers often utilize is the ratio of observations to parameters, in which a ratio of ten (our ratio was 15:143, falling close to that range) is deemed acceptable [45]. Initial examination of the data distribution revealed violations of normality assumptions. To adjust for potential biases, robust corrections of non-normality were conducted using the Satorra–Bentler scaled chi-squared statistic [46].

Specifically, there are two distinct steps in the modeling process of SEM with latent variables: structural and measurement modeling [42]. The structural model represents the main theory to be tested, which is that trauma history exacerbates depression and anxiety symptoms operating through sleep. The measurement portion of the model depicts the relationship between the factors (e.g., sleep) and the manifest variables that measure the factors (e.g., sleep hours, sleep onset latency, and nighttime awakenings). In our structural model, sleep was specified as the mediator, trauma as the main predictor, and depression and anxiety as the outcome variables. In terms of the measurement model, a latent construct of sleep was measured collectively by the subconstructs of sleep hours, sleep onset latency, and nighttime awakenings, all of which were collected and calculated using Fitbit watches.

To evaluate the performance of our model, fit indices were thoroughly examined, including Satorra–Bentler scaled chi-square results, the comparative fit index (CFI), standardized root mean square residual (SRMR), and root mean square error of approximation (RMSEA). Non-significant chi square, CFI > 0.95, SRMR < 0.08, and RMSEA < 0.06 would indicate well-fitting models based on Bentler’s recommendations [47].

## 3. Results

The sample was 53% female and 24% identified as White, 69% as Black, and 7% as biracial. These figures roughly represent the overall population served by the agency from which the sample was recruited. Fourteen percent of participants indicated obtaining a high school diploma and 85% reported having some type of employment. In regards to mental health measures, the mean score of the PHQ (depression) was 9.65 (SD = 3.68, Min = 3, Max = 21), the mean score of the BAI (anxiety) was 16.28 (SD = 9.26, Min = 1, Max = 45), and the mean score of the BTQ (trauma exposure) was 2.48 (SD = 2.40, Min = 0, Max = 8). The most commonly reported items were being severely physically punished by a parent or caregiver before age 18 (61% of the sample), being in a serious car accident (47%), and being attacked, beaten, or mugged by a friend or stranger (32%). As for the sleep measures, Fitbit-measured mean sleep hours were 5.61 (SD = 1.25, Min = 4, Max = 9.5), the mean onset latency time in minutes was 21.63 (SD = 10.36, Min = 4, Max = 54), and the mean number of nighttime awakenings was five (SD = 2.16, Min = 1, Max = 9).

Pearson’s correlations indicated that (1) trauma scores were significantly and positively associated with depression/anxiety, sleep onset latency, and nighttime awakenings and negatively correlated with sleep hours, and (2) sleep hours were significantly and negatively correlated with both depression and anxiety as well as the other two sleep indicators, while sleep onset latency and nighttime awakenings were positively associated with higher depression and anxiety (Table 1). The correlational patterns (between sleep and all mental health outcomes) confirmed the potential role of sleep in predicting mental health outcomes.

Initial descriptive statistics revealed no significant demographic (age, gender, education, and employment) differences in the scores of depression, anxiety, or trauma; hence, there was no need to add control variables. Multiple regression results indicated that all three mental health measures significantly negatively predicted sleep hours as measured by Fitbit.

### Multi-Level SEM

**Figure 1:**
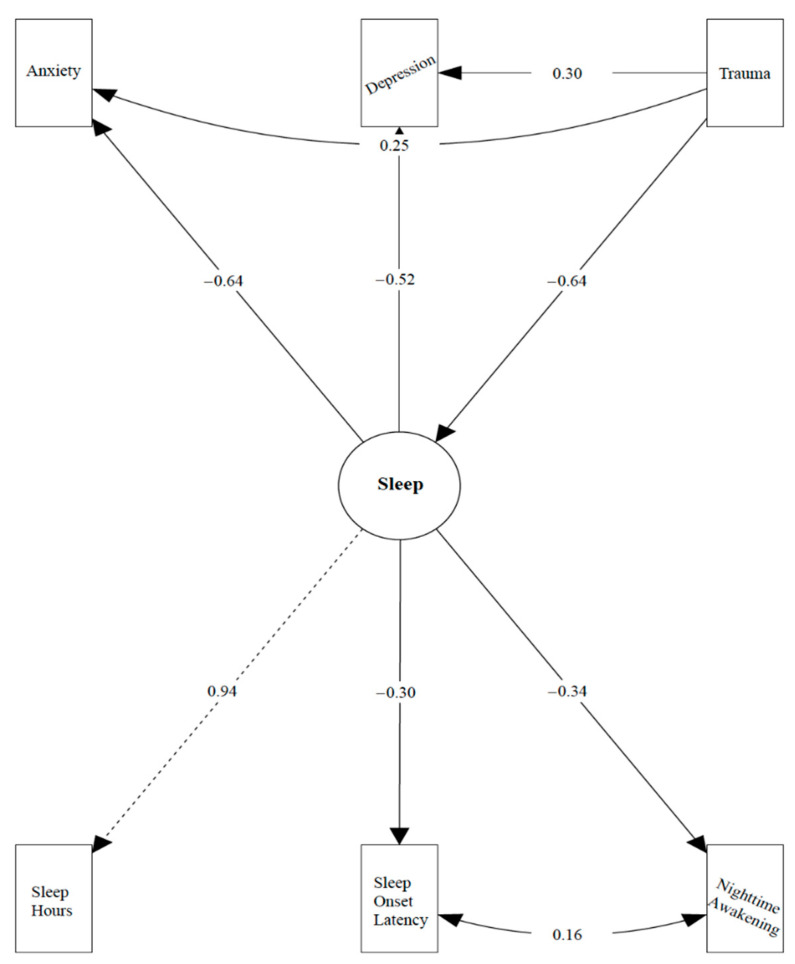
Discussion.

In Table 2, all path parameters of the SEM model are presented. The initial model had overall good fit, as evidenced by the following indices: *X*^2^ (5143) = 9.24, *p* = 0.1; CFI = 0.99; RMSEA = 0.077 (90% CI: 0.00, 0.154); and SRMR = 0.03. Informed by the measurement model, the factor structure of the latent construct “Sleep” was well identified. In general, the total effects (the direct and indirect paths from trauma to depression/anxiety) and indirect effects (from trauma to depression/anxiety, mediated by sleep) were both significant. Specifically, there existed (1) a direct negative prediction from trauma to individuals’ sleep quality (b = −0.32, z = −9.09, *p* < 0.001), a direct positive prediction from trauma to anxiety (b = 0.96, z = 2.15, *p* < 0.05), and from trauma to depression (b = 0.47, z = 2.81, *p* < 0.01); (2) a direct negative prediction from sleep to anxiety (b = −5.09, z = −3.98, *p* < 0.001), and from sleep to depression (b = −1.64, z = −3.65, *p* < 0.001); and (3) significant indirect effects (mediation) from trauma to both depression and anxiety. Given the significant direct effects after accounting for the indirect paths, sleep partially mediated the path from trauma to depression (b = −0.76, z = −5.25, *p* < 0.001) as well as to anxiety (b = −4.87, z = −3.78, *p* < 0.001) (Figure 1).

Young adults can be at risk for mental health problems due to social and environmental factors such as family adversity, peer stressors, and employment concerns [48]. Young adulthood is also the developmental period when mental health problems, especially mood disorders, may emerge or worsen [49]. Low-income young adults are more likely to report trauma exposure, which elevates risk for developing mental health problems [50]. Although sufficient and quality sleep promotes good health, a history of trauma and mental health problems can contribute to poor sleep.

Participants in the current study reported exposure to an average of 2.5 traumatic events, with the maximum number of events reported being eight. The most commonly reported items included both being severely punished as a child and being beaten, attacked, or mugged by a stranger or a friend. A study of adolescents with trauma histories found that each traumatic event experienced increased the odds of engaging in high-risk behaviors such as substance abuse, criminal activity, and self-injury by roughly 15% [11]. It is not only the number of traumatic events but also the type of trauma experienced that has an impact. Interpersonal trauma is significantly more likely to result in PTSD, especially severe intrusive memories, reminders of past trauma, and suppression of emotional responses, compared to experiencing non-interpersonal trauma [51]. A study of U.S. adults found that those who reported more than one type of traumatic event exposure, especially physical or sexual assault, were significantly more likely to have both PTSD and sleep problems [52].

We explored whether or not poor sleep plays a mediating role in mental health problems and, thus, a pattern evolves in which mental health problems contribute to poor sleep, and poor sleep exacerbates mental health problems. Our results showed that higher levels of trauma exposure were related to depression and anxiety. Fewer sleep hours partially mediated the relationship between both trauma exposure and depression and anxiety, and the direct effect from trauma remained significant after adjusting for the partial mediation from sleep.

The National Sleep Foundation [53] recommends 7–9 h of sleep per night for people aged 18 to 25. The young adults in the current study were receiving a mean total of 5.6 h, and some showed as few as four hours of sleep per night. Insufficient sleep in young adults has been linked to a range of negative outcomes including obesity, car accidents, and suicidal ideation [4]. A systematic review of studies revealed that youth who received less than six hours of sleep showed significant increases in health risk behaviors [54]. These relationships were stronger among young adults with emotional problems, poor psychosocial functioning, and a history of negative life events. Given the powerful effect that poor sleep can have on all aspects of functioning, especially among more vulnerable subpopulations, it is important to identify interventions to improve sleep.

One effective sleep intervention is Cognitive Behavioral Therapy for Insomnia (CBT-I). CBT-I targets the behavioral, cognitive, and physiological aspects of insomnia [55]. Multiple randomized control trials have shown the effectiveness of CBT-I in reducing sleep latency, increasing total sleep time, and decreasing nighttime awakenings [56]. Results of CBT-I have been found to be similar to or better than using sleep medications, and the maintenance of effects after cessation of therapy is better than with medications [57]. There is evidence that CBT-I may not only improve sleep but may reduce symptoms in people with anxiety, depression, or post-traumatic stress [58].

Young adults may also benefit from more easily accessible interventions. Education about good sleep hygiene such as having a regular sleep–wake schedule, avoiding caffeine and light-emitting devices close to bedtime, and getting regular exercise can be effective in improving sleep habits [59]. A study of college students found that those who attended an information session about the importance of sleep, the consequences of sleep deficits, and recommendations to improve sleep showed an increase of 30 min of total sleep time among those receiving less than seven hours of sleep before the program [60]. There is also growing evidence that activities such as meditation and yoga can improve sleep outcomes, even in adolescent populations [61].

Study results should be interpreted with some limitations in mind. First, while there is growing use of Fitbit monitors as a low-cost and easily accessible tool in sleep and health research, they may over- or underestimate sleep times. Second, assessments of traumatic events and childhood maltreatment were based on self-report. Memories of past events can be inaccurate, so young adults could be unintentionally misreporting events. Third, we cannot establish with certainty the direction of the relationship between sleep and trauma. Finally, the study was cross-sectional and only captured data at one point in time.

## 4. Conclusions

For individuals experiencing problems with depression and anxiety, improved sleep can serve as the gateway to better functioning. Low-income young adults, especially those who belong to marginalized groups, are much less likely to receive needed mental health services, not only due to finances but also because of fears about being judged or treated poorly [62]. Since poor sleep is much less stigmatized than mental health problems, people may be more likely to seek out treatment to assist with sleep concerns, which can then lead to improvements in mental health. Helping young adults get more and higher-quality sleep can increase overall health and well-being across the lifespan.

## Figures and Tables

**Table 1:** Simple correlations among trauma scores, depression/anxiety, and sleep indicators.

		1	2	3	4	5	6
1	Trauma	\					
2	Anxiety	0.66	\				
3	Depression	0.64	0.61	\			
4	Sleep Hours	−0.61	−0.75	−0.67	\		
5	Sleep Onset Latency	0.12	0.18	0.30	−0.29	\	
6	Nighttime Awakenings	0.15	0.31	0.22	−0.31	0.24	\

Note: All correlations were significant at the *p* < 0.05 level.

**Table 2:** Mediation model of trauma predicting depression and anxiety via mediation through sleep.

Measurement Model		B	SE	Z	P	Beta
Sleep	Sleep Hours	1.00				0.94
	Sleep Onset Latency	−2.66	0.79	−3.37	0.00	−0.30
	Nighttime Awakening	−0.62	0.17	−3.77	0.00	−0.34
**Structural Model**						
**Outcome**	**Predictor**					
Sleep	Trauma	−0.32	0.04	−9.09	0.00	−0.64
Anxiety	Trauma	0.96	0.45	2.15	0.03	0.25
Depression	Trauma	0.47	0.17	2.81	0.01	0.30
Anxiety	Sleep	−5.09	1.28	−3.98	0.00	−0.64
Depression	Sleep	−1.64	0.45	−3.65	0.00	−0.52
**Indirect and Total Effects**						
	Indirect Effect 1 (Anxiety)	−4.87	1.29	−3.78	0.00	−0.16
	Indirect Effect 2 (Depression)	−0.76	0.15	−5.25	0.00	−0.16
	Total Effect 1	−5.18	1.28	−4.04	0.00	−0.80
	Total Effect 2	−1.08	0.15	−7.40	0.00	−0.80

Fit indices: *X*^2^ (5143) = 9.24, *p* = 0.1; *CFI* = 0.99; *Root Mean Square Error of Approximation (RMSEA)* = 0.077 (90% CI: 0.00, 0.154); *Standardized Root Mean Residual (SRMR)* = 0.03. Path diagram of trauma. predicting depression/anxiety via sleep. B = Unstandardized beta; SE = Standard Error; Z = Z-scores; P = *p*-values.

## Data Availability

The data presented in this study are available on request from the corresponding author. The data are not publicly available due to guidelines of the Institutional Review Board overseeing the study.
